# Severe Recurrence of Neuroleptic Malignant Syndrome: Usefulness of Dexmedetomidine for Antipsychotic Withdrawal

**DOI:** 10.7759/cureus.47088

**Published:** 2023-10-15

**Authors:** Keisuke Maeda, Toshihisa Ichiba, Kenichiro Kashiwa, Yuji Okazaki

**Affiliations:** 1 Department of Emergency Medicine, Hiroshima Citizens Hospital, Hiroshima, JPN

**Keywords:** emergency medicine, recurrence of neuroleptic malignant syndrome, antipsychotic withdrawal, dexmedetomidine, neuroleptic malignant syndrome (nms)

## Abstract

Neuroleptic malignant syndrome (NMS) is a potentially fatal side effect that occurs in patients taking antipsychotics. Patients with NMS are often forced to rechallenge antipsychotic medications to control the underlying psychiatric symptoms. We present a case of severe recurrence of NMS in a patient in whom the administration of antipsychotics was restarted two days after NMS resolution. A 19-year-old man with somatic symptom disorder had been transported for fever, tachycardia, rigidity, and disturbance of consciousness. He was taking atypical antipsychotics with poor medication compliance. A diagnosis of NMS was made, and he was treated with administration of dantrolene sodium and benzodiazepines under tracheal intubation. On day 2, he was extubated. On day 4, his symptoms of NMS improved, but psychiatric symptoms rapidly exacerbated. He and his family strongly insisted on discharge, and we therefore unavoidably restarted the administration of antipsychotics. On day 37, he was retransported, and a diagnosis of recurrence of NMS was made. Blood examination showed marked deterioration of acute kidney injury and disseminated intravascular coagulation compared to those at the first admission. Without the administration of antipsychotics, his psychiatric symptoms were poorly controlled. Administration of dexmedetomidine helped his agitation to be well controlled without antipsychotics for two weeks. Short-term restart of antipsychotic drugs in patients with NMS may result in more severe NMS relapse. Dexmedetomidine may be useful for NMS patients when the administration of antipsychotics cannot be restarted. When antipsychotics are unavoidably rechallenged in patients with NMS, the risk of severe relapse should be taken into consideration, and dexmedetomidine may be used for prolongation of the withdrawal period.

## Introduction

Neuroleptic malignant syndrome (NMS) is a potentially fatal adverse event in patients taking antipsychotics [1]. There have been many reports on treatment for NMS. However, it is unclear when and how the administration of antipsychotics should be restarted for the underlying psychiatric disorders after the resolution of NMS. Also, the extent of severity of recurrence of NMS is not known [2,3]. We report a case of recurrent NMS with severe rhabdomyolysis, acute renal injury (AKI), and disseminated intravascular coagulation (DIC) after restarting the administration of antipsychotics two days after the resolution of NMS.

## Case presentation

A 19-year-old man with somatic symptom disorder had been transported for fever, rigidity, and disturbance of consciousness. He was taking olanzapine 20 mg daily, quetiapine 300 mg daily, and levomepromazine 150 mg daily for five months before admission. The doses of these drugs were not changed before admission. However, his medication compliance was poor and the exact amount of these drugs taken was unclear. He did not have a behavioral prodrome of several weeks that is characterized by psychosis, agitation, and catatonic excitement. He had diaphoresis and muscle rigidity in his extremities and neck, but no cataplexy. On arrival, Glasgow Coma Scale was 6 (E1V2M3), body temperature was 41.5 °C, blood pressure was 89/44 mmHg, heart rate was 173 beats per minute, respiratory rate was 50 times per minute, and oxygen saturation was 98% with administration of oxygen at 6 L/min. Blood examination revealed that creatine kinase (CK) was 1,384 U/L and creatinine was 1.50 mg/dL. Brain magnetic resonance imaging and lumbar puncture did not reveal the causes of his symptoms. We made a diagnosis of NMS and he was treated with administration of dantrolene sodium of 40 mg and sedated with continuous infusion of propofol under endotracheal intubation. On day 2, he was alert and was extubated. On day 4, his symptoms of NMS improved, but uncontrolled involuntary movements and psychiatric symptoms such as feeling of depersonalization rapidly exacerbated. These symptoms could not be controlled without antipsychotics before admission, and administration of benzodiazepines and biperiden was not effective. Psychiatrists considered that psychotherapy and patient education were necessary to control these psychiatric symptoms and that until these were performed, they could not be controlled without antipsychotics. Thus, based on his physical and mental status and response to these drugs, psychiatrists recommended to be committed to a mental institution, but he and his family strongly opposed the recommendation. Finally, we unavoidably restarted administration of olanzapine 20 mg and quetiapine 300 mg daily, and he was discharged to home on day 5.

**Figure 1:**
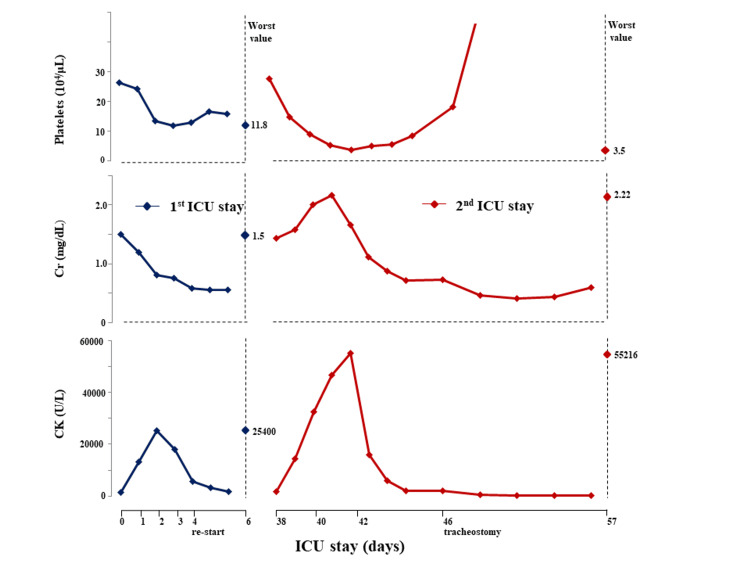
Clinical course The blue line shows the results of blood examinations at the first ICU admission, and the red line shows the results of the second ICU admission. The rightmost dots on the graphs show the worst values. All values were worse and the improvement took longer time in the second ICU admission than in the first ICU admission. ICU, intensive care unit; Cr, creatinine; CK, creatine kinase.

On day 37, he was transported to our emergency department again due to altered consciousness and muscle rigidity in his extremities. After the first discharge, his psychotic symptoms had been well controlled with antipsychotics. His symptoms and vital signs on arrival were almost the same as those at the first admission. Blood examination revealed that CK was 1,752 U/L and creatinine was 1.49 mg/dL. Based on his clinical presentation, well-controlled psychiatric symptoms including catatonia, and normal brain imaging and lumbar puncture, a diagnosis of recurrence of NMS rather than malignant catatonia was made. He was intubated and was sedated with high doses of benzodiazepine. After admission, blood examination showed marked deterioration of AKI and DIC compared to those at the first admission (Figure 1). He was successfully extubated on day 43. However, he had a fever, tachycardia, and respiratory failure with muscular fatigue induced by the repeated hyperinflammatory status of NMS. Thus, a tracheostomy was performed on day 46. His psychiatric symptoms such as agitation, hallucination, delusion, and catatonia were poorly controlled without antipsychotics. In addition, he could not be sedated with the usual dose of benzodiazepines because of the development of tolerance. Considering the risk of recurrence of NMS, he was mildly sedated with dexmedetomidine at the dose of 0.5-1.5 μg/kg/hour and was orally administrated lorazepam for catatonia. Dexmedetomidine was effective in achieving a well-controlled state of sedation and his psychiatric symptoms such as agitation and catatonia gradually improved over a two-week period. On day 60, sedation with continuous infusion of dexmedetomidine daily was no longer necessary. However, he was transferred to a mental institution on day 65 because his hallucination did not disappear after stopping the administration of dexmedetomidine.

## Discussion

Short-term restart of administration of antipsychotics in patients with NMS has two potential problems: 1) risk of recurrence of NMS and 2) possibility of the recurrence of NMS being more severe than the first episode. It is thought that the risk of recurrence is associated with the elapsed time between the extension of the withdrawal period and restarting the administration of antipsychotics, but there are few reports and no clear guidelines on the timing of the readministration of antipsychotics [4,5]. Although there are no clear recommendations regarding the timing of restarting antipsychotics in patients with NMS [6], it should be necessary to wait at least two weeks before restarting treatment in order to prevent the recurrence of NMS [7,8]. While early rechallenge of low dose and low potency of antipsychotics may be acceptable in unavoidable cases, a five-day withdrawal period after symptom resolution should be considered and close follow-up observation should be performed to prevent recurrence of NMS [9]. In our case, however, we had to resume the administration of antipsychotics only two days after the relief of symptoms of NMS due to the uncontrolled psychiatric symptoms and the strong demand for discharge from him and his family. As a result, our patient may suffer a recurrence of NMS, even though it was unavoidable. In addition, we should consider that recurrence of NMS in a short interval may be more severe than the first episode. Our patient's condition was complicated with more severe rhabdomyolysis with doubled CK, AKI, and DIC and with marked thrombocytopenia, and tracheostomy was required due to respiratory failure. Although there is little information on whether a short-term recurrence of NMS is more severe than the first episode, NMS is a potentially fatal condition and we should focus on preventing recurrence of NMS. Further studies are needed to clarify the relationship between time to relapse and the severity of NMS. It may also be necessary to investigate how the type and dose of antipsychotics contribute to the development, recurrence, and severity of NMS.

After the resolution of NMS, it is likely that there will be difficulty in controlling psychiatric symptoms in patients with psychiatric disorders without the administration of antipsychotics. Dexmedetomidine has often been used effectively for delirium in an intensive care unit, and it may be a useful alternative medication during the withdrawal period [10-12]. NMS is treated with sedatives such as benzodiazepines, but patients with psychiatric disorders have previous use of and tolerance to these drugs, which may make them less effective. Furthermore, catatonia, which must be differentiated from NMS, is also usually treated with benzodiazepines. However, if benzodiazepines are not effective for NMS and catatonia, electroconvulsive therapy may be required [13]. In contrast, dexmedetomidine does not acquire tolerance because of no previous use, and there is no risk of developing NMS. Moreover, unlike benzodiazepines, dexmedetomidine can also be used after extubation without adversely affecting the respiratory status. Even when it is impossible to restart administration of antipsychotics in the acute phase of NMS or to administer pre-NMS doses of the agents, dexmedetomidine may be considered to be used to sedate NMS patients with psychiatric disorders and to stabilize agitation in order to prolong the withdrawal period for prevention of recurrence of NMS. However, this benefit has been only reported as a case report. Thus, further studies are needed to investigate the efficacy and safety of dexmedetomidine for antipsychotic withdrawal in patients with NMS.

## Conclusions

Patients with NMS require intensive care management, and not only intensivists but also psychiatrists are often involved in treatment. However, there is still no established management for how to safely restart antipsychotics and how to ensure a withdrawal period before administering them in patients with NMS. To summarize this case, a short-term restart of antipsychotics in patients with NMS may lead to a more severe relapse of NMS, so we recommend ensuring a withdrawal period of antipsychotic administration as long as possible after the symptoms of NMS are resolved. In addition, if we have difficulty controlling psychiatric symptoms in patients with NMS, dexmedetomidine may be useful to extend the withdrawal period.
